# Differentiating the effect of antipsychotic medication and illness on brain volume reductions in first-episode psychosis: A Longitudinal, Randomised, Triple-blind, Placebo-controlled MRI Study

**DOI:** 10.1038/s41386-021-00980-0

**Published:** 2021-02-26

**Authors:** Sidhant Chopra, Alex Fornito, Shona M. Francey, Brian O’Donoghue, Vanessa Cropley, Barnaby Nelson, Jessica Graham, Lara Baldwin, Steven Tahtalian, Hok Pan Yuen, Kelly Allott, Mario Alvarez-Jimenez, Susy Harrigan, Kristina Sabaroedin, Christos Pantelis, Stephen J. Wood, Patrick McGorry

**Affiliations:** 1 Turner Institute for Brain and Mental Health, School of Psychological Sciences, Monash University, Clayton, VIC Australia; 2 Monash Biomedical Imaging, Monash University, Clayton, VIC Australia; 3 Orygen, Parkville, VIC Australia; 4 Centre for Youth Mental Health, The University of Melbourne, Melbourne, VIC Australia; 5 Melbourne Neuropsychiatry Centre, Department of Psychiatry, University of Melbourne & Melbourne Health, Melbourne, VIC Australia; 6 The Florey Institute for Neuroscience and Mental Health, University of Melbourne, Melbourne, VIC Australia; 7 Department of Social Work, Monash University, Clayton, VIC Australia; 8 Melbourne School of Population and Global Health, University of Melbourne, Parkville, VIC Australia; 9 School of Psychology, University Birmingham, Edgbaston, UK

**Keywords:** Psychosis, Psychosis

## Abstract

Changes in brain volume are a common finding in Magnetic Resonance Imaging (MRI) studies of people with psychosis and numerous longitudinal studies suggest that volume deficits progress with illness duration. However, a major unresolved question concerns whether these changes are driven by the underlying illness or represent iatrogenic effects of antipsychotic medication. In this study, 62 antipsychotic-naïve patients with first-episode psychosis (FEP) received either a second-generation antipsychotic (risperidone or paliperidone) or a placebo pill over a treatment period of 6 months. Both FEP groups received intensive psychosocial therapy. A healthy control group (*n* = 27) was also recruited. Structural MRI scans were obtained at baseline, 3 months and 12 months. Our primary aim was to differentiate illness-related brain volume changes from medication-related changes within the first 3 months of treatment. We secondarily investigated long-term effects at the 12-month timepoint. From baseline to 3 months, we observed a significant group x time interaction in the pallidum (*p* < 0.05 FWE-corrected), such that patients receiving antipsychotic medication showed increased volume, patients on placebo showed decreased volume, and healthy controls showed no change. Across the entire patient sample, a greater increase in pallidal grey matter volume over 3 months was associated with a greater reduction in symptom severity. Our findings indicate that psychotic illness and antipsychotic exposure exert distinct and spatially distributed effects on brain volume. Our results align with prior work in suggesting that the therapeutic efficacy of antipsychotic medications may be primarily mediated through their effects on the basal ganglia.

## Introduction

Magnetic resonance imaging (MRI) has been used extensively to document brain changes in psychotic disorders. Grey matter volume (GMV) reductions relative to healthy controls are particularly robust, and evident across all illness stages [1–3] and in multiple brain regions [2, 4, 5]. Some of these changes appear to worsen with transition to psychosis and ongoing illness [6], which has been taken as evidence of a progressive process associated with illness onset [7], although some have opposed this view [8, 9].

Numerous mechanisms have been proposed to explain longitudinal brain changes in schizophrenia, including aberrant neurodevelopment [10], neuroinflammation [11], network-based pathological spread [12], and the iatrogenic effects of antipsychotic treatment [4, 13]. Widespread and early treatment of patients with antipsychotics has made it notoriously difficult to disentangle the effects of medication and pathophysiology on brain volume. Although studies of antipsychotic-naïve patients clearly show brain GMV reductions in the absence of medication [2], several lines of evidence suggest that antipsychotic medication influences GMV [14]. For example, longitudinal studies suggest that cumulative exposure to antipsychotic medication is associated with reduced total cerebral [13] and prefrontal GMV [4], and studies in macaques and rodents have shown that chronic exposure to first- and second- generation antipsychotics reduces total GMV [15, 16] and glial cell number [17]. One recent placebo-controlled trial in mostly remitted patients with psychotic depression showed that, compared to patients on placebo, those given olanzapine had decreased cortical thickness within both hemispheres [18].

Other work suggests that antipsychotic medication, particularly second-generation antipsychotics, may exert a neuroprotective effect [19]. Studies in rodents have supported a neuroprotective effect of second-generation antipsychotics [20], which may arise through several candidate mechanisms, including neurogenesis [19] and protection against oxidative stress [21]. This work parallels naturalistic [22] and experimental [19] longitudinal MRI studies in human patients suggesting that second-generation antipsychotics may be associated with less GMV loss when compared to first-generation antipsychotics.

**Fig. 1:**
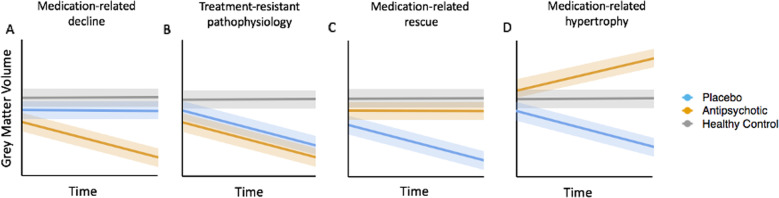
Disentangling illness-related and antipsychotic medication-induced brain changes in early psychosis using a randomised placebo-controlled design. Each panel presents a schematic of expected results under different hypotheses. **A** A medication-related decline due to antipsychotics is indicated if medicated patients show accelerated GMV loss compared to patients in the placebo group and healthy controls. **B** An illness effect that is not modified by treatment is indicated if both treatment groups show accelerated GMV loss relative to controls. **C** An illness-related change that is rescued by antipsychotics is indicated if GMV loss is observed in the placebo group but not medicated patients. **D** Antipsychotic-related hypertrophy, where GMV is increased in the medicated group compared to the healthy controls and/or placebo group, could be consistent with either a possible medication-related rescue or the initial stages of a volume-loss process (e.g. an oedemic reaction). These possibilities could be disentangled by examining correlations with symptomatic or functional measures; e.g. an association between the volumetric increase and improved outcome would be consistent with possible rescue. For simplicity, controls are depicted as showing no change over time, but they may also show longitudinal increases or decreases. The key factor is whether the rate of change is greater in patients compared to controls. Solid lines represent group means and shaded areas represent some estimate of the error around the mean.

One limitation affecting all existing longitudinal studies conducted thus far is that they have only examined patients who are receiving antipsychotic medication. This approach is problematic because previous exposure to medication may result in brain changes that could mask or be mistaken for illness-related processes. The only way to unambiguously distinguish illness-related from medication-related brain changes is through a randomised placebo-controlled study of antipsychotic-naïve first-onset patients, in which one patient group is exposed to antipsychotic medication and the other receives a placebo. This design is able to test several distinct hypotheses about the differential contributions of illness and antipsychotics to brain changes in the earliest illness stages (Fig. 1). However, such experiments are difficult to conduct due to the practical difficulties and ethical concerns associated with withholding antipsychotic treatment.

We recently overcame these challenges to conduct, to our knowledge, the first randomised, triple-blind, placebo-controlled trial of antipsychotic medication in first-episode psychosis (FEP), in which antipsychotic-naïve patients were randomised to receive psychosocial therapy with or without antipsychotic medication over the first 6 months of treatment engagement [23]. We found, using a non-inferiority design [23], that the placebo group showed comparable clinical and functional outcomes to the medicated group at the end of the treatment study [24]. Here, we report an analysis of GMV in this cohort, where MRI was acquired before treatment (baseline), at 3 months, and then at a 12-month follow-up. Our primary aim was to distinguish volumetric changes attributable to illness from those attributable to antipsychotic medication within the initial 3-month period (Fig. 1). Our secondary aim was to investigate longer-term changes at the 12-month follow-up, after a period of time in which some subjects in the placebo group had been exposed to antipsychotics. We also examined whether any observed volumetric changes were associated with symptomatic and functional changes.

## Method

### Study design

**Fig. 2:**
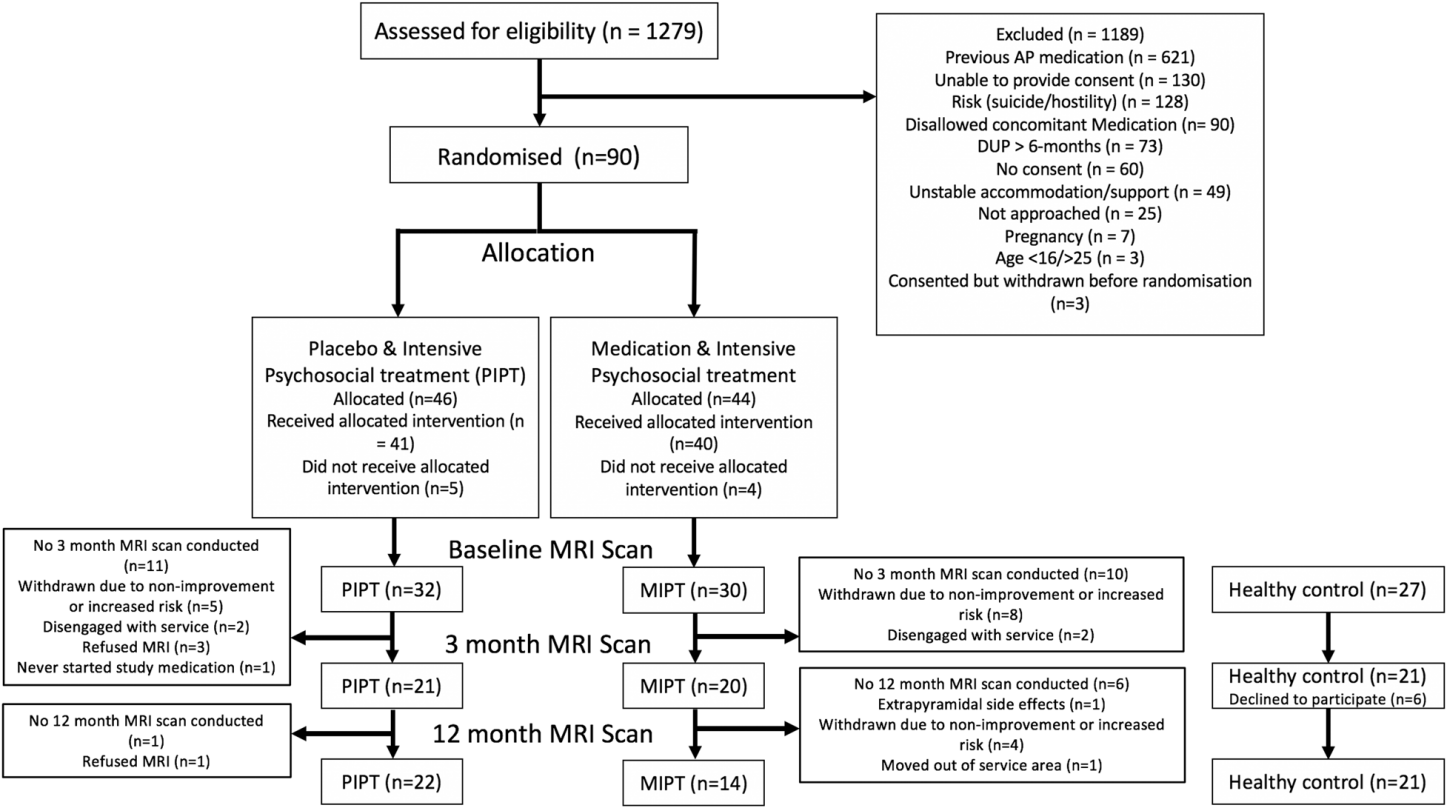
Recruitment diagram for the study. The flow of patients and healthy contol participants though the study.

Patients were randomised to one of two groups: one given antipsychotic medication plus intensive psychosocial therapy (MIPT) and the other given a placebo plus intensive psychosocial therapy (PIPT) (Fig. 2). A third healthy control group who received no intervention was also recruited. For both patient groups, the treatment period spanned 6 months. MRI and clinical assessments were conducted at baseline, 3 months, and a final follow-up at 12 months. The randomisation phase of the study terminated at 6 months, so patients in either the MIPT or PIPT group could have received antipsychotic medication and ongoing psychosocial interventions in between the 6 and 12 months into the study. Further research and safety protocols can be found in the Supplement and elsewhere [23]. Ethical approval for the study was granted by the Melbourne Health Human Research Ethics Committee (MHREC:2007.616).

### Participants

Patients were aged 15–25 years and were experiencing a first-episode of psychosis, defined as fulfilling Structured Clinical Interview for DSM-5 (SCID) criteria for a psychotic disorder, including schizophrenia, schizophreniform disorder, delusional disorder, brief psychotic disorder, major depressive disorder with psychotic symptoms, substance-induced psychotic disorder or psychosis not otherwise specified. Additional inclusion criteria to minimise risk were: ability to provide informed consent; comprehension of English language; no contraindication to MRI scanning; duration of untreated psychosis (DUP) of less than 6 months; living in stable accommodation; low risk to self or others; minimal previous exposure to antipsychotic medication (<7 days of use or lifetime 1750 mg chlorpromazine equivalent exposure; further details provided in Supplementary Table 1).

The aim of this study was to include individuals with FEP, which by definition, can include multiple diagnostic groups, due to the diagnostic heterogeneity and instability in the early stages of psychotic illness. We adopt this transdiagnostic approach given that antipsychotics are not a specific treatment for schizophrenia alone but are recommended as a first line treatment for all first-episode psychoses, evidence that clinical outcomes do not differ between substance-induced and other first-episode psychoses [25], and that similar neurobiological changes, including in the striatum, have been noted in patients with psychosis that cut across traditional diagnostic categories [26–28].

Healthy control participants were aged between 18 and 25, could provide written informed consent, and were psychiatrically, neurologically and medically healthy. Current and historic psychiatric illness was ruled out using self-report, the SCID and The Comprehensive Assessment of At-Risk Mental States [29]. In addition, participantes were excluded if they were currently taking any psychotropic medications. A stratified randomisation design, with Gender and DUP as factors, was used to allocate patients to either MIPT or PIPT treatment groups. DUP was included as a three-level factor (0–30, 31–90, and >90 days). Clinicians, patients, study assessors and researchers conducting MRI pre-processing remained blinded to treatment allocation throughout the trial. Further details on inclusion criteria, safety measures, and discontinuation criteria can be found elsewhere [23]. A recruitment flow diagram and final group numbers at each timepoint are presented in Fig. 2 and demographic are presented in Table 1.

**Table 1:** Sample characteristics and group differences at baseline.

	First-episode psychosis		Healthy control (*N* = 27)	*T*/*χ*^2^ (*p*)^a^
	PIPT (*N* = 30)	MIPT (*N* = 29)		
Baseline age, years (SD)	18.8 (2.72)	19.5 (2.94)	21.9 (1.93)	−4.49 (<0.001)
Females, *N* (%)	14 (46.6%)	13 (44.8%)	17 (62.9%)	2.19 (0.139)
Handedness, left, *N* (%)	2 (6.7%)	2 (6.9%)	3 (11.1%)	0.465 (0.495)
Education, years (SD)	11.8 (1.86)	12.7 (2.30)	15.2 (1.90)	−6.21 (0.001)
Diagnosis, *N*				
Major depression with psychosis	7	5	–	
Schizophreniform disorder	5	5	–	
Psychotic disorder NOS	8	7	–	
Substance-induced psychotic disorder	4	2	–	
Delusional disorder	1	4	–	
Schizophrenia	5	5	–	
Missing diagnosis	0	1	–	
Baseline BPRS total, mean (SD)	59.4 (9.64)	55.8 (10.10)	–	1.40 (0.166)
Baseline SOFAS, mean (SD)	52.9 (14.0)	51.7 (10.6)	–	0.384 (0.703)
Baseline SANS, mean (SD)	37.3 (17.2)	32.9 (18.1)	–	0.961 (0.346)
Baseline HAM-D, mean (SD)	19.5 (6.75)	18.2 (6.57)	–	0.745 (0.459)
Baseline HAM-A, mean (SD)	22.2 (6.42)	20.2 (7.25)	–	1.118 (0.268)
Baseline QLS, mean (SD)	68.5 (24)	70.6 (20.8)	–	−0.368 (0.714)

*PIPT* placebo plus intensive psychosocial therapy, *MIPT* antipsychotic medication plus intensive psychosocial therapy, *NOS* not otherwise specified, *BPRS* Brief Psychiatric Rating Scale version 4, *SOFAS* Social and Occupational Functioning Assessment Scale, *SANS* Scale for the Assessment of Negative Symptoms, *HAM-D* Hamilton Depression Rating Scale, *HAM-A* Hamilton Anxiety Rating Scale, *QLS* Quality of Life Scale.

^a^This column provides the *T* or *χ*^2^ values comparing the healthy control and patients (collapsed across two treatment conditions) at baseline.

### Symptomatic and functional measures

The preregistered primary and secondary trial outcome measures were the total Social and Occupational Functioning Assessment Scale (SOFAS) and the BPRS-4 scores, respectively [23]. Additional measures can be found in Supplementary materials.

### Antipsychotic medication

Patients randomised to the MIPT group received either 1 mg risperidone (*n* = 25) or 3 mg paliperidone (*n* = 5). To reflect real-world clinical treatment, this starting dose was then increased according to clinical response by the blinded treating clinician. The same procedure was followed for participants in the PIPT group, who received a placebo pill that was identical in taste, appearance, and packaging to the active medication. Additional details can be found in Supplementary materials.

### MRI acquisition and pre-processing

A 3-T Siemens Trio Tim scanner located at the Royal Children’s Hospital in Melbourne, Australia, was used to acquire a high resolution structural T1-weighted scan for each participant. The MRI data were processed using the Computational Anatomy Toolbox and Diffeomorphic Anatomical Registration Exponentiated Lie algebra algorithm [30]. Additional details can be found in Supplementary materials.

### Statistical analyses

Mixed-effects marginal models were used to analyse regional GMV across the three groups (MIPT, PIPT and Healthy control) and three timepoints (baseline, 3 months and 12 months follow-up). The models were implemented at voxel-level in the Sandwich Estimator Toolbox [31] (version 2.1.0). All other statistical analyses were conducted in R-studio (version 1.1.423).

Our primary analyses sought to disentangle the effects of medication and illness (e.g. Fig. 1) on total GMV and to map localised changes using VBM. This analysis focused on the baseline and 3-month timepoints, as they fell within the treatment period. For both total and regional GMV, we first tested for baseline differences between groups using an analysis of covariance, controlling for age at baseline, sex, handedness and total intracranial volume. We then examined longitudinal changes using a marginal model. The voxel-level analysis was implemented in the Sandwich Estimator Toolbox [31] (version 2.1.0), which uses ordinary least squares estimators of group-level regression parameters and a modified sandwich estimator for standard errors [32]. This method allows for inclusion of subjects with missing MRI data at any timepoint, by robust and accurate estimation of random effects while also mitigating problems posed by mis-specification of covariance structure when using traditional mixed-effects models [31]. The contrast of interest was a group (MIPT, PIPT, Control) by time (baseline, 3 months) interaction. We performed inference using non-parametric bootstrapping (10,000 bootstraps), with statistical significance for total volume assessed at *p* < 0.05, and a *p* < 0.05, family-wise error (FWE)-corrected threshold for voxel-level analyses, as implemented in the Sandwich Estimator Toolbox [31]. To provide a more complete picture, we also report results surviving a less stringent threshold of *p* < 0.001, uncorrected, with an extent threshold of ten voxels, but caution that these findings require replication. Uncorrected voxel-level statistical maps are available at: https://neurovault.org/collections/9346/. Our secondary analysis included the 12-month timepoint and was designed to examine the long-term effects of early withholding of antipsychotic medication. Similar procedures were used as in the primary analysis. Details of the 12-month analysis and of analyses addressing symptom correlates and confounding variables are in the Supplementary material.

## Results

### Demographics and clinical characteristics

There were no significant differences between the patient and control samples in sex or handedness, but the patients were, on average, 1.9 years younger and had 2 years less education (Table 1). At baseline, the two patient groups (PIPT and MIPT) did not significantly differ in age, education, sex, handedness, BPRS or SOFAS score.

### Baseline differences in total and regional grey matter volume

No significant baseline difference in total GMV was detected between patients (collapsed across treatment groups) and healthy control participants (*F* = 0.297; *p* = 0.588), nor were any voxel-level regional differences detected following whole-brain FWE-correction. Results at an uncorrected threshold can be found in Supplementary Table 3. As expected, no significant differences in total GMV were detected between the two patient groups at baseline (*F* = 0.352, *p* = 0.555).

### Disentangling medication-related and illness-related brain changes in the first 3 months of treatment

**Fig. 3:**
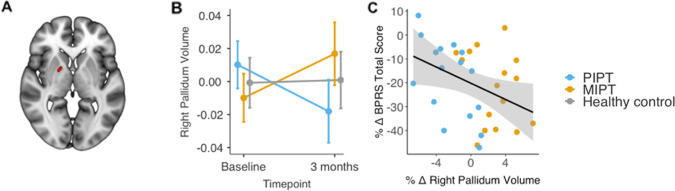
Primary Findings (Baseline to 3-months). **A** Red = Location of the cluster within the right pallidum where a significant group × time interaction (*p* < 0.05, FWE-corrected) was detected. **B** The principal pallidal GMV eigenvariate for each group at baseline and 3-month follow-up, adjusted for model covariates. Error bars show 95% confidence intervals. **C** The association between percentage change (%Δ) in total Brief Psychiatric Rating Scale score (BPRS; *y*-axis) and percentage change in pallidal GMV volume within the two treatment groups.

No significant group by time interaction in total GMV was detected between the three groups (*F* = 0.387, *p* = 0.689). Using VBM to map regional changes, a significant group by time interaction was identified within the right pallidum (*p* < 0.05, FWE-corrected; Fig. 3a). From baseline to 3 months, GMV in this region remained stable in controls, decreased in the PIPT group, and increased in the MIPT group (Fig. 3b). The primary post hoc contrasts revealed that, compared to baseline, pallidal GMV significantly decreased in PIPT patients (*t* = 2.34, *p* = 0.021), increased in MIPT patients (*t* = −2.20, *p* = 0.029), and did not change in controls (*t* = −0.142, *p* = 0.888). This result indicates that the interaction is driven by both an increase in the MIPT group and a decrease in the PIPT group over time. Secondary post hoc contrasts conducted at the 3-month timepoint showed that the PIPT had significantly less pallidal GMV than the MIPT (*t* = −2.26, *p* = 0.012), with neither group differing from controls.

Greater increase in pallidal GMV over 3 months was associated with a greater reduction in symptom severity, as indexed by the BPRS Total score (*ρ* = −0.418; *p* = 0.017; Fig. 3c). There was no significant association with SOFAS total score (*ρ* = −0.002; *p* = 0.998) and none of the exploratory correlations with ancillary clinical measures survived correction for multiple comparisons (Supplementary Table 4). At an uncorrected threshold, we found a negative correlation between right pallidal volume and BRPS positive symptom change scores that was comparable in magnitude to the association with BPRS total (*ρ* = −0.431; *p* = 0.012), suggesting that the relationship between pallidal volume and symptom change may be specifically related to positive symptoms. Results at an uncorrected threshold (*p* < 0.001) can be found in the Supplementary Fig. 2.

### Potential confounds

No statistically significant associations were found between percentage change in pallidal volume from baseline to 3 months and DUP, concomitant medication use, or substance use. Details are in the Supplementary materials.

### Disentangling medication-related and illness-related brain changes in the first 12 months of treatment

We next considered MRI measures at the 12-month follow-up to differentiate the long-term effects of medication and illness. Patients retained at the 12-month follow-up did not significantly differ in baseline age (*t* = 1.7858, *p* = 0.08), sex (*χ*^2^ = 0.087, *p* = 0.767), education (*t* = 0.652, *p* = 0.518), BPRS (*t* = −0.076, *p* = 0.940) or SOFAS (*t* = 0.780, *p* = 0.439) from those who did not complete the 12-month follow-up scan. In addition, patients retained at the 12-month follow-up did not significantly differ at 3 months in age (*t* = 1.71, *p* = 0.120), sex (*χ*^2^ = 0.028, *p* = 0.867), education (*t* = 1.14, *p* = 0.285), BPRS (*t* = 0.001, *p* = 0.999) or SOFAS (*t* = −0.710, *p* = 0.488) from those who did not complete the 12-month follow-up scan.

No statistically significant differences in linear trend for total GMV over the 12-month follow-up period were detected (*F* = 1.60, *p* = 0.192).

At the voxel-level, no statistically significant regional differences were identified at the FWE-corrected threshold. Results at an uncorrected threshold (*p* < 0.001) can be found in the (Supplementary Fig. 3).

In addition, we assessed whether the changes seen within the pallidal cluster and the correlation with clinical symptoms, which we identified in the primary analysis, persisted at 12-month follow-up. The differences between the three groups were not statistically significant (Supplementary Fig. 4) and volume change was not associated with the primary trial outcome measures. However, we found a negative correlation between change in pallidal volume and BRPS positive symptom change scores at 12 months (*ρ* = −0.50; *p* = 0.001). This result aligns with our findings at 3 months and suggests that the relationship between pallidal volume and symptom change may be specifically related to positive symptoms.

### Assessing the specificity of findings to grey matter

To assess the specificity of our findings to grey matter, we repeated the above primary and secondary analyses in white matter. For the primary analysis, we found a significant group by time interaction within a small area of left cerebellar lobule IX white matter (*k* = 9, *p* < 0.05, FWE-corrected; Supplementary Fig. 5a). From baseline to 3 months, white matter volume in this region increased in the controls (*t* = −2.34, *p* = 0.021), remained stable in the PIPT group (*t* = 0.216, *p* = 0.830), and decreased in the MIPT group (*t* = 2.239, *p* = 0.027). This pattern of results is consistent with medication-related volume loss (e.g. Fig. 1a). The change in volume within this cluster was not correlated with change in BPRS-4 or SOFAS. In addition, volume change from baseline to 3 months in the cerebellar white matter cluster was negatively correlated with volume change in the pallidal cluster (*ρ* = −0.466; *p* value = 0.006). Results at an uncorrected threshold can be found in the Supplementary material.

## Discussion

We used a triple-blind, placebo-controlled randomised trial to disentangle the effects of medication and illness on GMV change within early stages of first-episode psychosis. We found evidence of regionally heterogeneous effects associated with both illness and medication, with the most robust effect being an illness-related decline of pallidal GMV in the placebo group coupled with an antipsychotic-related increase in the medicated group. Consistent with a therapeutic benefit of the antipsychotic-induced increase in pallidal GMV, a greater volumetric change in this area was associated with a greater reduction in symptomology within the first 3 months of illness. Evidence for medication-related white matter decline was identified in the cerebellum. These results suggest that both psychotic illness and medication exposure exert distinct and spatially distributed effects on GMV, and converge with prior work in suggesting that the therapeutic efficacy of antipsychotic medications is primarily mediated through their effects on the basal ganglia [33, 34].

### Illness-related volumetric reductions in FEP

Pallidal volume in the PIPT group declined over the first 3 months of illness. This decline was not associated with substance use or concomitant medication, which would be consistent with an illness-related effect. In contrast, MIPT patients showed an increase in pallidal volume over time. Thus, antipsychotic medication appears to prevent or perhaps even reverse illness-related volume loss in this part of the brain.

Using less stringent criteria for significance, we found evidence for illness-related GMV reductions in visual cortex within the first 3 months of illness, and further reductions in prefrontal cortex GMV and white matter by the 12-month timepoint. These changes were observed in both the PIPT and MIPT groups, which is consistent with an unmodified effect of illness (Fig. 1b), but these results require replication as they did not survive whole-brain correction.

The pallidum is the primary output structure of the striatum, and disturbances of fronto-striato-thalamic circuits have long been implicated in the pathogenesis of psychosis [28]. The function of these circuits is heavily modulated by dopamine, and their disruption is apparent in diagnosed patients [26], patients’ unaffected first-degree relatives [26] and individuals experiencing an at-risk mental state for psychosis [27]. Functional connectivity within this circuit also correlates with the severity of psychotic-like experiences in non-clinical samples [35]. Thus, one hypothesis that may explain our findings is that altered signalling from the striatum triggers early volumetric loss in the pallidum [28, 36], which subsequently spreads to affect functionally related prefrontal areas [37, 38]. This interpretation aligns with evidence that smaller pallidal volume in antipsychotic-naïve patients is associated with more severe psychiatric symptomology [39], and our own finding that increased pallidal volume over the first 3 months correlates with improved symptom outcome. Moreover, our finding of possible long-term reductions in prefrontal cortex grey and white matter may explain why prefrontal dysfunction is so commonly reported in patients with established illness. However, the precise mechanisms underlying volumetric changes in psychosis remain a topic of debate [8, 9]. Here, we show that some of these changes cannot be attributed to medication but other confounding factors such as differences in hydration, physical and mental activity, and stress cannot be ruled out. Targeted mechanistic studies are required before we can draw strong inferences about pathophysiological mechanisms.

### Are antipsychotics neuroprotective?

The increase of pallidal volume seen in MIPT patients, together with the correlation between increased pallidal GMV and symptom improvement between baseline and 3 months, are consistent with a putative neuroprotective effect of second-generation antipsychotic medication (Fig. 1d). Larger pallidal volumes have been widely reported in medicated [40] but not antipsychotic naïve [41] patients, whereas larger volume in other basal structures, such as the putamen, have been reported irrespective of medication status [42]. Human studies have also shown reduced volume loss in patients taking second-generation antipsychotics compared to patients receiving first-generation antipsychotic medication [19], and work in animals indicates that second-generation antipsychotics can exert several neuroprotective effects [20], including induction of neurogenesis [43], and protection against oxidative stress [21], as well as positive effects on cognition [44]. Our results are in line with this work and suggest that second-generation antipsychotics prevent illness-related volume loss occurring early in the illness. However, MRI is unable to identify a specific cellular mechanism that would support a neuroprotection hypothesis, and the molecular mechanisms by which second-generation medications might protect grey matter structures in humans are poorly understood. Second-generation antipsychotics are characterised by relatively high affinities for both serotonin and dopamine receptors. Patients in our study received risperidone or its molecularly similar active metabolite paliperidone. Both medications are antagonists for 5HT_2_ receptors in addition to showing high affinity for D_2_ receptors [45]. Rodent studies using risperidone have demonstrated cell proliferation [46], increased levels of brain-derived neurotrophic factor [47], and the promotion of antioxidant defence [48]. Thus, while our data suggest that antipsychotics may rescue or perhaps reverse illness-related decline of pallidal volume within the first 3 months of illness, and that this apparent preservation of volume is associated with improved symptom outcomes, further studies are required to elucidate underlying cellular and molecular mechanisms.

Pallidal volume had normalised in both MIPT and PIPT patients by 12 months (Supplementary Fig. 4). While it is possible that this normalisation reflects differences in illness characteristics between patients who did and did not complete the 12 months follow-up, we found no significant difference in baseline or 3-month demographic and clinical characteristics between these two groups. Nonetheless, it is possible that patients completing the 12-month assessment followed distinct illness trajectories after enrolment into the study. An alternative explanation is that early pallidal changes reflect an acute illness effect with subsequent normalisation reflecting a compensatory process. It is also possible that adminstration of a placebo pill and/or intensive psychosocial therapy and engagement with clinical services, was sufficient to normalise volumes [49] in the PIPT group. Volume normalisation in the MIPT group may also reflect a plastic adaptation that returns volumes to normal levels after an initial response to treatment. Whether these volume changes reflect differences in synaptic proliferation, cell size/density or tissue perfusion and/or hydration remains an open question [9, 50].

### Evidence of medication-related decline in grey matter volume

We found no evidence for antipsychotic-related decline in GMV at whole-brain-corrected thresholds. At the less stringent uncorrected threshold, we observed consistent medication-related GMV reductions in the cerebellum at 3 months and at 12 months (Fig. 1a), but results should be replicated before they can be considered robust. Similar results surviving whole-brain correction were observed in cerebellar white matter. While previous naturalistic [4] and experimental [18] studies have demonstrated an association between antipsychotic medication and loss of both total and hemispheric volume, our study is distinct in several important ways. First, our study included a placebo control and healthy control, allowing us to experimentally isolate the effect of second-generation antipsychotic medication. Second, we examined patients during a relatively short time span of 1 year, while other studies have examined longer periods [4]. Third, the mean cumulative dosage of antipsychotic medication in our study, while still an effective dose [51], is considered low. Fourth, all participants in our study were scanned with the same scanner, mitigating the potentially confounding effects of scanner differences. Finally, all patients within our study received an evidence-based psychosocial intervention which may have associated neuroprotective effects [49]. In light of these differences, our results could be interpreted as preliminary evidence of potential neurotoxicity in early illness stages that is predominantly expressed in the cerebellum. However, there was no association between volume change within the cerebellum and change in functional or symptom outcome scores, in either grey or white matter. Longer-term follow-up would be required to determine the extent to which further volume-loss emerges with additional antipsychotic exposure.

### Relation to non-inferiority clinical trial findings

This MRI study took place in the context of a larger clinical trial where we found that the PIPT group showed non-inferior clinical and functional outcomes to the MIPT group at the end of the treatment study [24]. There are important differences between the clinical non-inferiority trial and the results reported here. First, the clinical trial examined the primary outcome at 6 months, whereas the current study’s primary outcome point is at 3 months. It is therefore possible that antipsychotic medication may be superior in the very early stages of treatment, but non-inferior when compared to 6 months of psychosocial treatment. Second, the sample used in the current study and the clinical trial are not identical due to differing rates of participation and attrition. Finally, while our findings indicate that medication increases pallidal volume, and that this increase is associated with better symptom outcome, it is possible that psychosocial intervention has clinical benefits that are not reflected in brain volume. Thus, antipsychotics and psychosocial intervention may both have positive effects on symptom reduction, with the former being better reflected in brain volume.

### Strengths and limitations

The strengths of this study include a prospective randomised control trial design, antipsychotic-naïve patients, triple blinding to treatment, and the inclusion of a healthy control group as a reference for characterising normative change over time. We also used robust non-parametric inference to model longitudinal changes in GMV [31]. In order for this study to satisfy ethical concerns, our inclusion criteria meant that patients who entered the study posed low risk of harm to self or others, lived in stable accommodation, and had a short DUP. In addition, patients who did not improve in clinical symptomology or functioning were removed from the trial, which contributed to attrition. It is therefore possible that our final patient cohort represents a sub-sample of individuals with a distinct form of psychotic illness characterised by reduced safety concerns and a potentially less progressive form of illness. As such, the illness-related changes we report here may be a conservative estimate of those that would be observed in a more heterogeneous sample that includes patients with greater risk levels and a potentially more progressive form of illness. Conversely, the homogeneity of our sample may have enhanced our ability to identify medication-related brain changes. We also note that the mean baseline SOFAS scores of our patients were comparable to epidemiologically representative cohorts of FEP patients [52] and that the mean baseline BPRS score of patients in our study (57.6) would classify them as ‘markedly ill’ [53]. In addition, there were no differences in baseline clinical or demographic characteristics between patients who did and did not complete the study. Thus, prima facie, there are few obvious differences between our cohort and many FEP samples reported in the literature, beyond the strict safety requirements of our study. Nonetheless, we cannot rule out the possibility that patients who remained in the study have a form of psychotic illness that is perhaps less severe and/or progressive than those who did not complete. In addition, compared to the PIPT group there was higher attrition in the MIPT group between the 3- and 12-month timepoints. For completeness, we report secondary results at an uncorrected statistical threshold and thus caution that these need to be replicated before they can be considered robust. Another possible limitation is that we did not directly examine the potential impact of concomitant antidepressant medication. However, the percentage of each treatment group who received antidepressant medication did not differ between the two treatment groups. A final limitation is that we only examined risperidone and paliperidone. It remains to be seen whether our results generalise to other antipsychotic medications.

## Conclusion

Taken together, our results demonstrate that psychotic illness and antipsychotic exposure exert distinct and spatially distributed effects on brain volume, with the most robust effect being consistent with an antipsychotic-related rescue of pallidal volume changes in the early stages of treatment.

## Funding and disclosure

Janssen-Cilag partially supported the early years of this study with an unrestricted investigator-initiated grant and provided risperidone, paliperidone and matched placebo for the first 30 participants. The study was then funded by an Australian National Health and Medical Research Project grant (1064704). The funders had no role in study design, data collection, data analysis, data interpretation, or writing of this report. The corresponding author had full access to all of the data in the study and had final responsibility for the decision to submit for publication. In the past 5 years, CP served on an advisory board for Lundbeck, Australia Pty Ltd. He has received honoraria for talks presented at educational meetings organised by Lundbeck. The authors have declared that there are no other conflicts of interest in relation to the subject of this study.

## Supplementary information

SUPPLEMENTAL MATERIAL
