# Supplementary material for: Differentiating the effect of antipsychotic medication and illness on brain volume reductions in first-episode psychosis: A Longitudinal, Randomised, Triple-blind, Placebo-controlled MRI Study
Source: Neuropsychopharmacology. 2021 Feb 26;46(8):1494–501. doi: 10.1038/s41386-021-00980-0 (PMC8209146; doi:10.1038/s41386-021-00980-0)
Supplement: Supplementary file 1 — SUPPLEMENTAL MATERIAL [file 41386_2021_980_MOESM1_ESM.docx]

Supplementary Materials

**Study Design and Funding – Additional Details**

The trial took place at the Early Psychosis Prevention and Intervention Centre, which is part of Orygen Youth Health, Melbourne, Australia. The trial was registered with the Australian New Zealand Clinical Trials Registry in November 2007 (ACTRN12607000608460) and received ethics approval from the Melbourne Health Human Research and Ethics committee.

Role of Funding Sources: Janssen-Cilag partially supported the early years of this study with an unrestricted investigator-initiated grant and provided risperidone, paliperidone and matched placebo for the first 30 participants. The study was then funded by an Australian National Health and Medical Research Project grant # 95757. The funders had no role in study design, data collection, data analysis, data interpretation, or writing of this report. The corresponding author had full access to all of the data in the study and had final responsibility for the decision to submit for publication.

**Additional Clinical and Functional Measures**

Additional measures of clinical and functional included the Scale for Assessment of Negative Symptoms (SANS), Hamilton Depression Scale (HAM-D) and Hamilton Anxiety Scale (HAM-A), and the World Health Organisation Quality of Life Scale -Brief (WHOQoL-BREF). Substance use was measured using the World Health Organisation Alcohol, Smoking and Substance Involvement Screening Test (WHO-ASSIST). Duration of untreated psychosis was measures using clinical interview.

**Antipsychotic and Concomitant Medication**

Participants allocated to the medication group received a starting dose of risperidone (1 mg) or paliperidone (3 mg), depending on when they were enrolled in the study, as the availability of a matched placebo pill varied. Dosing was titrated upwards depending upon clinical response, by the prescription of additional tablets/capsules after medical review appointment with blinded treating psychiatrist. At each phase of the study, placebo group participants received placebo tablets that were identical in appearance, taste, and packaging to the active medication. Other medications, excluding other antipsychotic medication and mood stabilizers, were permitted during the trial. Concomitant medications were permitted during the trial, except for additional antipsychotics or mood stabilisers (rates of concomitant medications use are provided in Supplementary Table 2). Four patients within the PIPT group were switched to open-label antipsychotic medication before the 3-month MRI scan and were excluded from the primary analysis. After termination of the randomization phase at 6 months, five patients in the PIPT group were exposed to antipsychotic medication between the 3-month and 12-month scan. We examined the impact of this exposure in our analysis of the 12-month data, as detailed below. Mean cumulative dose and rates of exposure for both patient groups at each timepoint are provided in [Supplementary Table 1](#SupplementaryTable1). Duration of untreated psychosis (DUP) was assessed using a clinical interview.

**MRI Acquisition and Pre-processing**

A 3-T Siemens Trio Tim scanner located at the Royal Children's Hospital in Melbourne, Australia, was used to acquire a high resolution structural T1-weighted Magnetisation-Prepared Rapid Gradient Echo (MPRAGE) scan for each participant. Image acquisition parameters at each timepoint were as follow: 176 sagittal slices, with a 1mm^3^ voxel size, bandwidth 236 Hz/pixel, FOV=256×256 mm, matrix 256×256, 2300 ms repetition time, and 2.98 echo time and a 9 -degree flip angle.

Prior to pre-processing, all raw T1w images were visually examined for artefacts and then subjected to an automated quality control procedure^5^. Four patient MRI scans did not pass image quality control and were excluded due to excessive head movement (n=1) or image artefacts (n=3). The remaining scans were pre-processed using the longitudinal pipeline of the Computational Anatomy Toolbox^6^ (version r1113) for the Statistical Parametric Mapping 12 (SPM12) software^7^ running in Matlab version 2015a. Briefly, for each participant, the T1w images from all available timepoints were rigidly realigned to correct for differences in head position within-subject, and a subject-specific mean image was calculated and used as a reference in a subsequent realignment of all T1w images across all timepoints. The mean image was segmented into grey matter, white matter, and cerebrospinal fluid, and normalised using the Diffeomorphic Anatomical Registration using Exponentiated Lie algebra algorithm (DARTEL)^7^. The resulting spatial normalisation parameters were then applied to the segmentation of the bias-corrected individual images for all available timepoints, and the resulting native space segmentation was used to calculate overall total intracranial volume and total GMV volume (ml) using FSLstats. The grey matter images were then again realigned to a DARTEL normalised template. Finally, the voxel level intensities were modulated by both the linear and non-linear Jacobian determinants derived from the previous spatial normalisation to preserve the total amount of grey matter. Finally, the resulting modulated and normalised grey matter images were spatially smoothed using an 8mm Gaussian kernel.

**Statistical Analysis - Additional Details**

Our secondary analysis included the 12-month follow-up timepoint, with the contrast of interest was a linear polynomial contrast examining differences in linear trend between the three groups. We constrain our contrasts in this way, because our hypotheses concern linear interactions between group and time over the follow-up period (as per [Figure 1](#Figure1)). The treatment period for the trial ended after 6-months. Thus, in principle, clinicians and patients were no longer bound by the treatment protocol after this point. In practice, four PIPT patients commenced antipsychotic medication in this intervening period, in addition to the four patients who had commenced at the 3-month timepoint. Thus, between the 3-month and 12-month scan, a total of eight PIPT patients commenced antipsychotics, whereas all MIPT patients continued medication with varying degrees of exposure. We thus specified a covariate quantifying cumulative exposure to antipsychotics (olanzapine equivalent, milligrams) for all eight patients within the PIPT group who were exposed to medication at the 3-month or 12-month timepoint. This procedure allowed us to statistically adjust for antipsychotic exposure in the PIPT group when attempting to disentangle the long-term effects of illness and medication exposure on GMV. To ensure that this approach did not substantially influence the results, we repeated the analysis after removing the eight antipsychotic-exposed patients from the PIPT group. The results were largely consistent (see Supplementary Figure 1).

We investigated the potentially confounding effects of DUP, concomitant medication, and substance use on GMV change in each region showing a significant group x time interaction. The effect of DUP prior to recruitment was examined using (1) a one-way ANOVA to examine baseline differences between three DUP strata (0-30 days, 31-90 days, and >90 days); and (2) a two-way ANOVA to assess whether there was a significant interaction effect between time, DUP stratum, and treatment group. To study the effects of non-antipsychotic medications (see [Supplementary Table 2](#SupplementaryTable2)), we conducted a three separate two-way ANOVA testing for an interaction between time and percentage patients who received each of the three classes of concomitant medication (benzodiazepines, antidepressants and other psychotropic medication) during the treatment period (the “other” category included people taking zopiclone, dexamethasone, benztropine and clonidine). To assess the effect of substance use, we ran Spearman correlations between regional GMV change and either the WHO-ASSIST total substance use score or the cannabis use sub-score.

We assessed the functional impact of any regions showing a statistically significant group by time interaction by correlating percent change in GMV over time (measured by the first eigenvariate of the region, adjusted for covariates) with percent change in scores on the pre-registered^8^ primary outcomes of the clinical trial; namely the SOFAS and BPRS-4 total scores. Associations were quantified using Spearman's rank correlations with the threshold for significance set at p<.025 (Bonferroni-adjusted for two comparisons). Additional exploratory correlations between all available clinical and functional scales were Bonferroni-corrected for ten comparisons (p<.005).

**Results of the Baseline Analysis at an Uncorrected Threshold**

At an uncorrected threshold (k > 10, p < 0.001; [Supplementary Table 3](#SupplementaryTable3)), patients showed reduced GMV within the right postcentral gyrus, right supramarginal gyrus, right frontal pole, right insula, middle temporal gyrus, and left hippocampus. As expected, no significant total (F = 0.530, p = 0.470) or voxel-level GMV differences were found between the MIPT and PIPT groups at baseline at either corrected or uncorrected thresholds.

**Results of the Baseline to 3 Months Analysis at an Uncorrected Threshold**

At an uncorrected threshold (k > 10, p < 0.001), we identified interactions between group and time that were consistent with a unmodified illness-related effect within lateral occipital cortex ([Supplementary Figure 1a](#Figure4), [Figure 1b](#Figure1)); a medication-related decline within cerebellum ([Supplementary Figure 1b](#Figure4), [Figure 1a](#Figure1)); and medication-related hypertrophy within the inferior temporal cortex, precuneus, and orbitofrontal cortex ([Supplementary Figure c-e](#Figure4), [Figure 1d](#Figure1)).

**Results of the Baseline to 12 Months Analysis at an Uncorrected Threshold**

At p < 0.001 (k=10) uncorrected, there were differences in linear trend consistent with unmodified illness-related changes ([Figure 1b](#Figure1)) within the bilateral dorsolateral superior frontal gyrus ( [Supplementary Figure 3a-b](#Figure5)), right superior orbito-frontal gyrus ([Supplementary Figure 3c](#Figure5)), middle orbito-frontal gyrus ([Supplementary Figure 3d](#Figure5)), and left superior medial frontal gyrus ([Supplementary Figure 3e](#Figure5)); a medication-related decline ([Figure 1a](#Figure1)) within the right cerebellar crus I ([Supplementary Figure 3f](#Figure5)); and medication-related hypertrophy ([Figure 1d](#Figure1)) within the right middle temporal gyrus ([Supplementary Figure 3g](#Figure5)), temporal pole ([Supplementary Figure 3h](#Figure5)) and cerebellar VIII ([Supplementary Figure 3i](#Figure5)). The results were largely consistent when eight individuals within the PIPT group who were exposed to antipsychotic medication between the 3- and 12-month scans were removed from the analysis (Supplementary Figure 2).

**Results of the Confounding Variables Analyses**

The effect of DUP on baseline pallidal volume was not significant (F = .011, p = .918), nor was the interaction between DUP and treatment group on percentage change in pallidal volume between baseline and 3 months (F = .240; p = .628). Similarly, the interactions between treatment group and use of benzodiazepines (F = 1.01; p = .359), antidepressants (F =.552; p = .463) or other psychotropic medication (F = 2.05; p = .163) on percentage change in pallidal volume between baseline and three months were not significant. We also found no significant correlation between percentage change in pallidal volume and total substance use (ρ = .049; p =.786) or cannabis use (ρ = -.186; p = .300). Additionally, further examination into the potential confounding effects of benzodiazepine use found that the time (baseline to 3-months) by use of benzodiazepines interaction on pallidal volume was not significant (F=2.007; p = .106), nor was a time by group by use of benzodiazepines interaction (F=.157; p = .692).

Supplementary Table 1 – Cumulative antipsychotic exposure (in Olanzapine milligram equivalates)

|  | Baseline | 3-months | 12-months |
| --- | --- | --- | --- |
| PIPT, mg (M, SD) | 0.16 (0.59) | 78.5 (216)^1^ | 608 (1111) |
| MIPT, mg (M, SD) | 1.03 (5.57) | 420 (248) | 1311 (1011) |

^1^ Note: In our primary analysis, the 4 patients within the placebo (PIPT) group who were exposed to antipsychotic medication at amounts greater than the study inclusion criteria at the 3-month timepoint were excluded from the analysis, thus cumulative antipsychotic exposure of the analysis sample was 4.57mg (14.5)

Supplementary Table 2 – Percentage of each treatment group who received each class of concomitant medication between baseline and 3-months

|  | Benzodiazepine | Antidepressant | Other^1^ |
| --- | --- | --- | --- |
| PIPT, % | 30.0 | 56.7 | 30.0 |
| MIPT, % | 62.0 | 51.7 | 41.4 |

^1^ This category included people taking zopiclone, dexamethasone, benztropine and clonidine.

Supplementary Table 3 – Regions showing reduced grey matter volume in patients at baseline at an uncorrected threshold (p < .001)

| *Baseline*  *Comparison* | Hemisphere | | Peak MNI_spm_  coordinates (x,y,z) | Cluster size voxels (mm^3^) |
| --- | --- | --- | --- | --- |
| Supramarginal gyrus | | Right | 52.5, -36, 37.5 | 191 (645) |
|  | |  |  |  |
| Hippocampus | | Left | -31.5, -28.5, -12 | 94 (317) |
|  | |  |  |  |
| Middle-Temporal cortex | | Right | 49.5, -58.5, 3 | 93 (314) |
|  | |  |  |  |
| Frontal pole | | Right | 13.5, 60, 7.5 | 57 (192) |
|  | |  |  |  |
| Postcentral gyrus | | Right | 18, -36, 48 | 30 (101) |
|  | |  |  |  |
| Insula Cortex | | Right | 31.5, 15, -1.5 | 21 (71) |

Supplementary Table 4 – Correlations between percentage change in secondary clinical scales and percentage change in pallidal GMV volume within the two treatment groups, from baseline to 3months.

| *Scale* | Rho (ρ) | *p* |
| --- | --- | --- |
| BPRS-positive | -.431 | .012 |
| SANS-total | -.044 | .807 |
| HAM-D | -.340 | .053 |
| HAM-A | -.190 | .290 |
| WHO-QoL | -.247 | .181 |

BPRS-positive = Brief Psychiatric Rating Scale version 4, positive symptoms subscale; SANS = Scale for the Assessment of Negative Symptoms; HAM-D = Hamilton Depression Rating Scale; HAM-A = Hamilton Anxiety Rating Scale; WHO-QLS = World Health Organisation - Quality of Life scale. Note: Quality of Life Scale (QLS) was not collected at the 3-month timepoint.


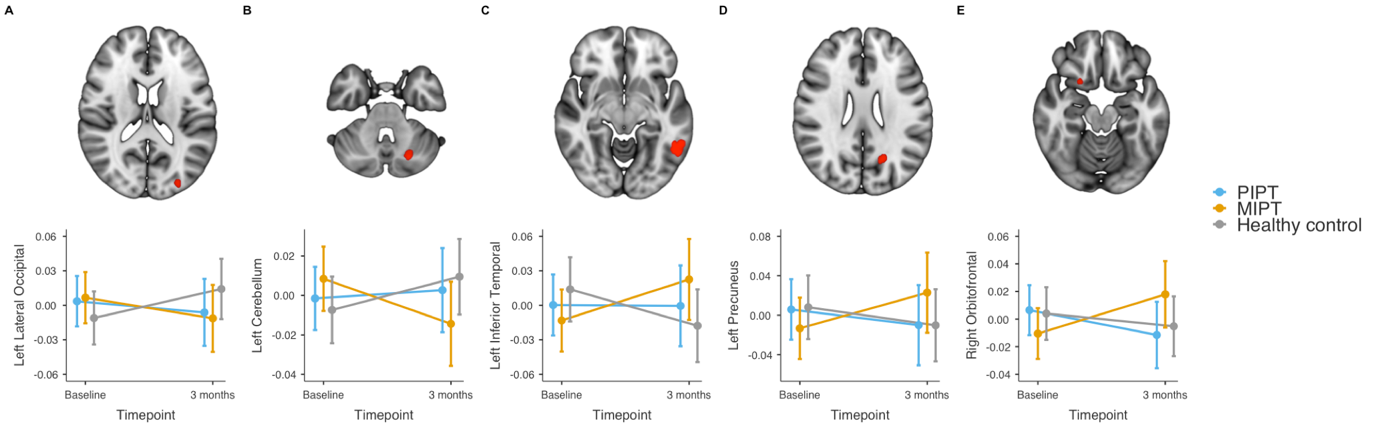


Supplementary Figure 1 – Red clusters indicate anatomical locations where significant group by time interactions (p < 0.001, uncorrected) were detected between baseline and 3 months. Bottom row shows the nature of the interaction. A) left lateral occipital cortex; B) left cerebellum; C) left inferior temporal cortex; D) left precuneus; E) right orbitofrontal. Abbreviations: PIPT = placebo plus intensive psychosocial therapy, MIPT = antipsychotic medication plus intensive psychosocial therapy.


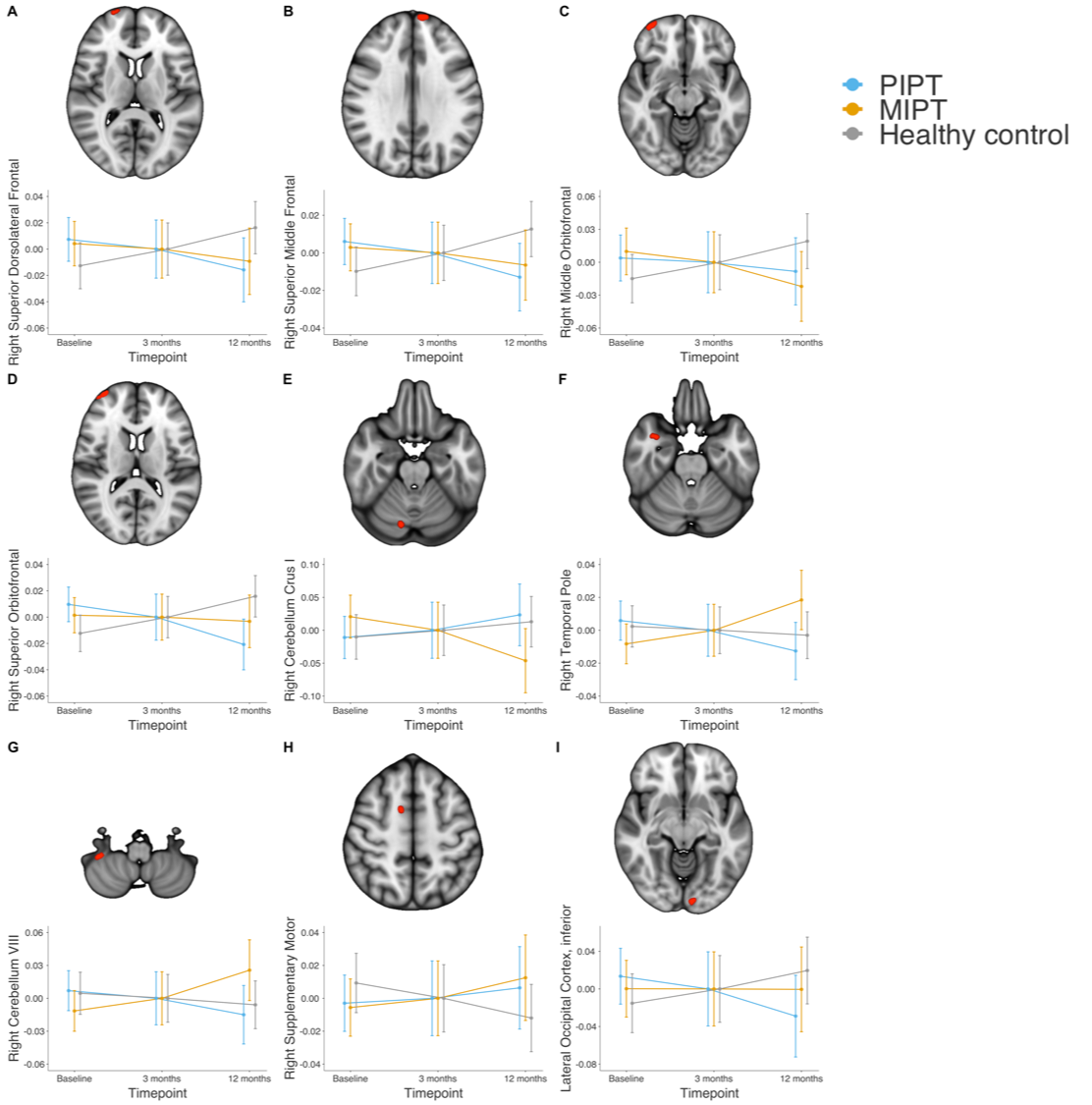


Supplementary Figure 2 – Red clusters indicate anatomical locations where differences in linear trend (p < 0.001, uncorrected) were detected between baseline and 12 months. In this analysis, patients in the placebo group who were exposed to antipsychotic medication during the 12 months were removed from the analysis. Bottom row of each panel shows the nature of the interaction. Abbreviations: PIPT = placebo plus intensive psychosocial therapy, MIPT = antipsychotic medication plus intensive psychosocial therapy.


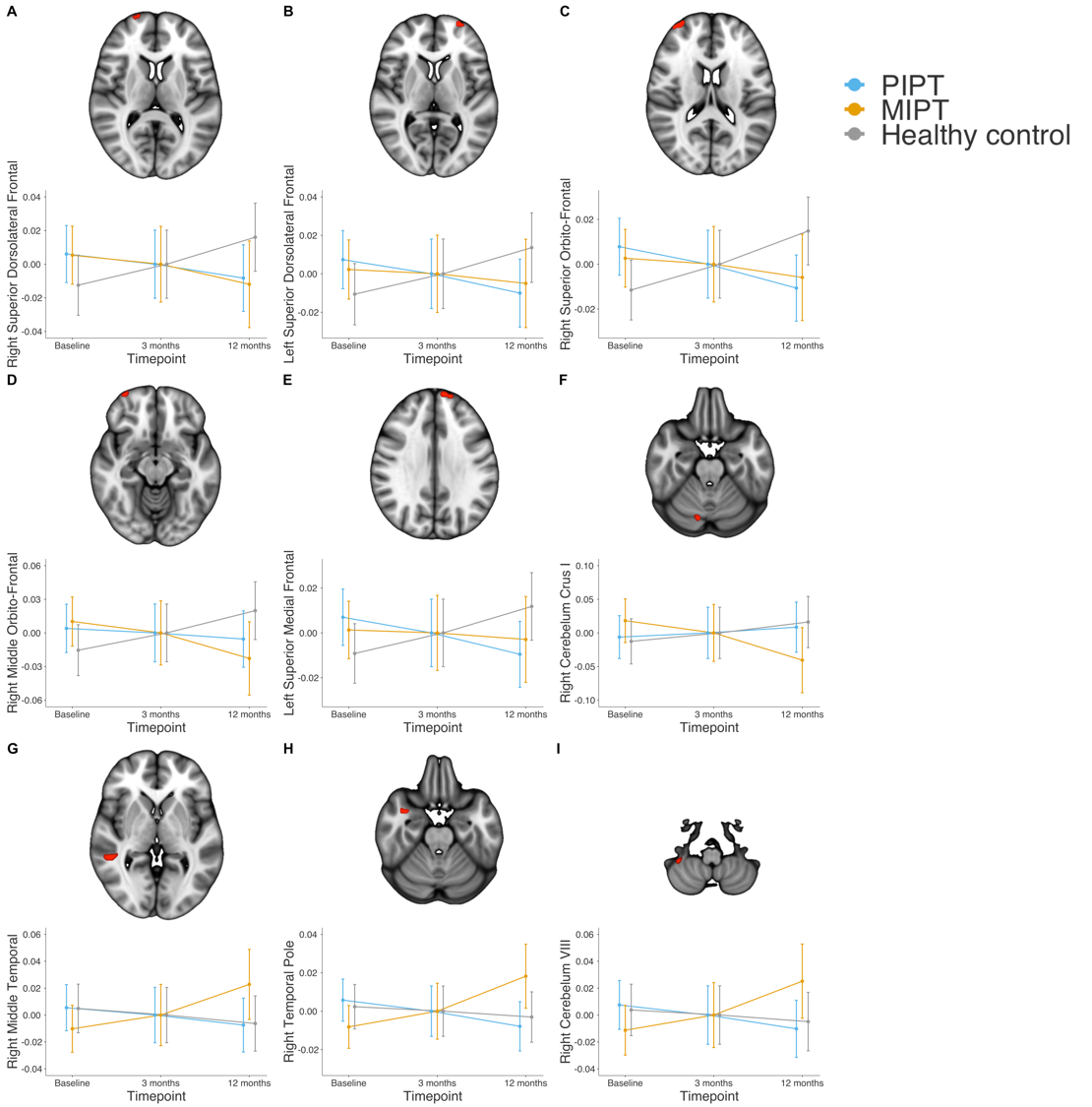


Supplementary Figure 3 – Red clusters indicate anatomical locations where differences in linear trend (p < 0.001, uncorrected) were detected between baseline and 12 months. Bottom row of each panel shows the nature of the interaction. A) right dorsolateral superior frontal gyrus; B) left dorsolateral superior frontal gyrus; C) right superior orbito-frontal gyrus; D) right middle orbito-frontal gyrus; E) left superior medial frontal gyrus; F) right cerebellar crus I; G) right middle temporal gyrus; H) right temporal pole; I) right cerebellar VIII; Abbreviations: PIPT = placebo plus intensive psychosocial therapy, MIPT = antipsychotic medication plus intensive psychosocial therapy.


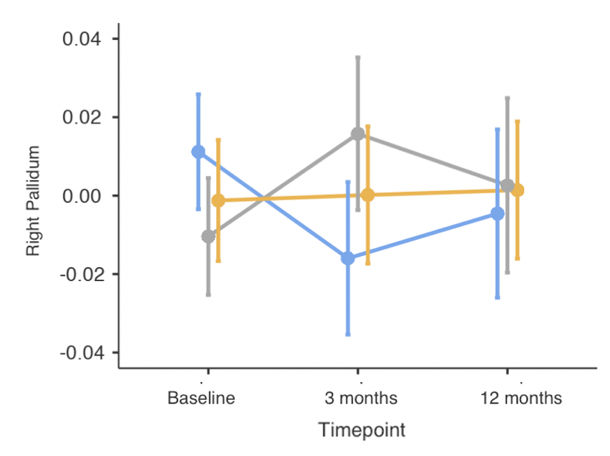

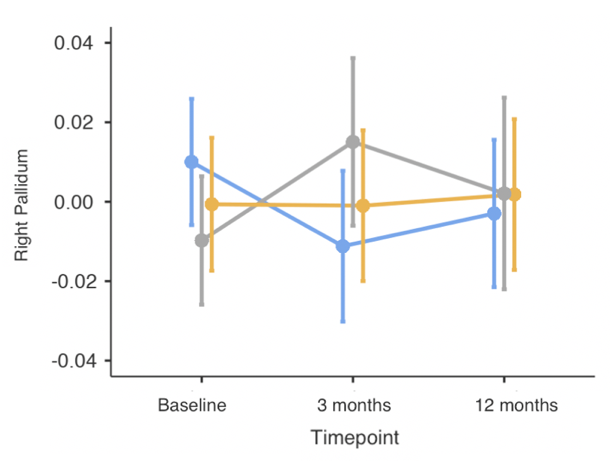


B

A

Supplementary Figure 4 – Volume of pallidal cluster at 12-months follow-up. A) The principal pallidal GMV eigenvariate for each group at baseline and 3-month follow-up, and 12-month follow-up, adjusted for model covariates. B) The pattern of results remained largely the same after patients in the PIPT group who were exposed to antipsychotic medication were removed from the analysis.

**Results from White Matter Analyses at an Uncorrected Threshold**

At an uncorrected threshold (k > 10, p < 0.001), interaction effects were detected within the white matter of the right cerebellar lobule V and crus II. These effects were consistent with a putative neurotoxic effect and an unmodified illness-related change, respectively ([Supplementary Figure 5b-c](#SupplementaryFigure3)).

No significant interactions were detected when including the 12-month time point. At an uncorrected threshold (k > 10, p < 0.001), an interaction effect consistent with a unmodified illness-related change was detected within left frontal white matter (Supplementary Figure 6).


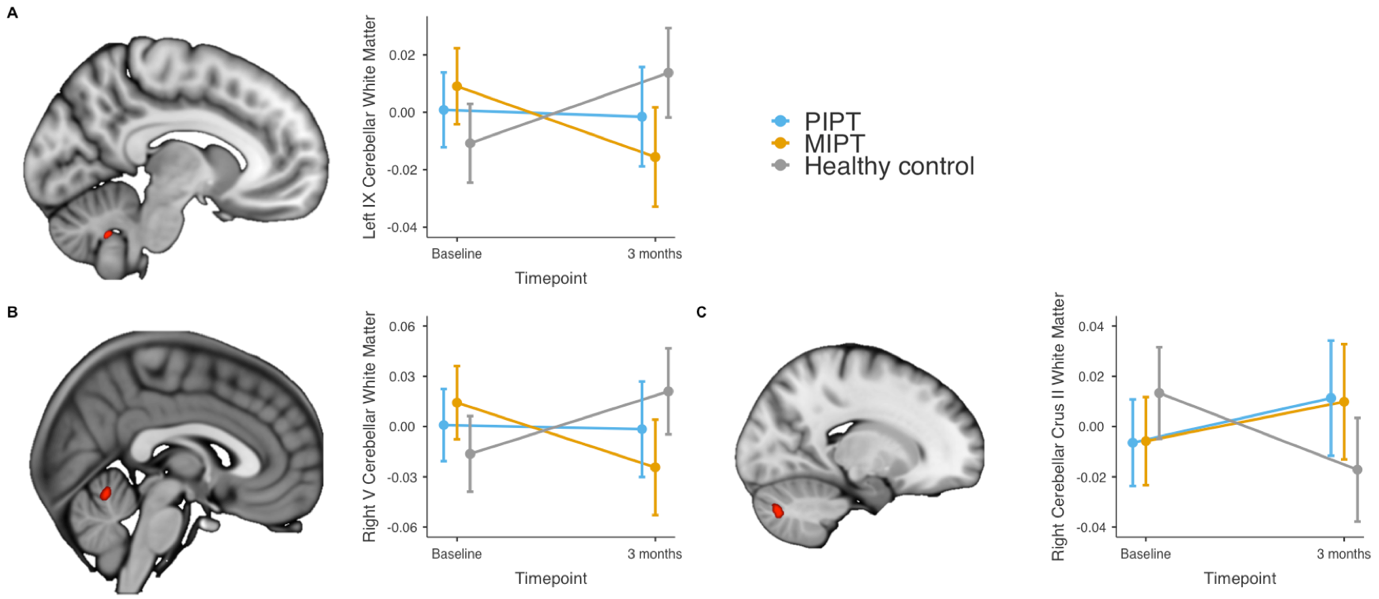


Supplementary Figure 5 – The principal cerebellar white matter volume eigenvariate for each group at baseline and 3-month follow-up, adjusted for model covariates. Error bars show 95% confidence intervals.

A) Cerebellar lobule IX cluster (p < 0.05, FWE-corrected) B) cerebellar lobule V (p < 0.001, uncorrected), C) Cerebellar crus II (p < 0.001, uncorrected)


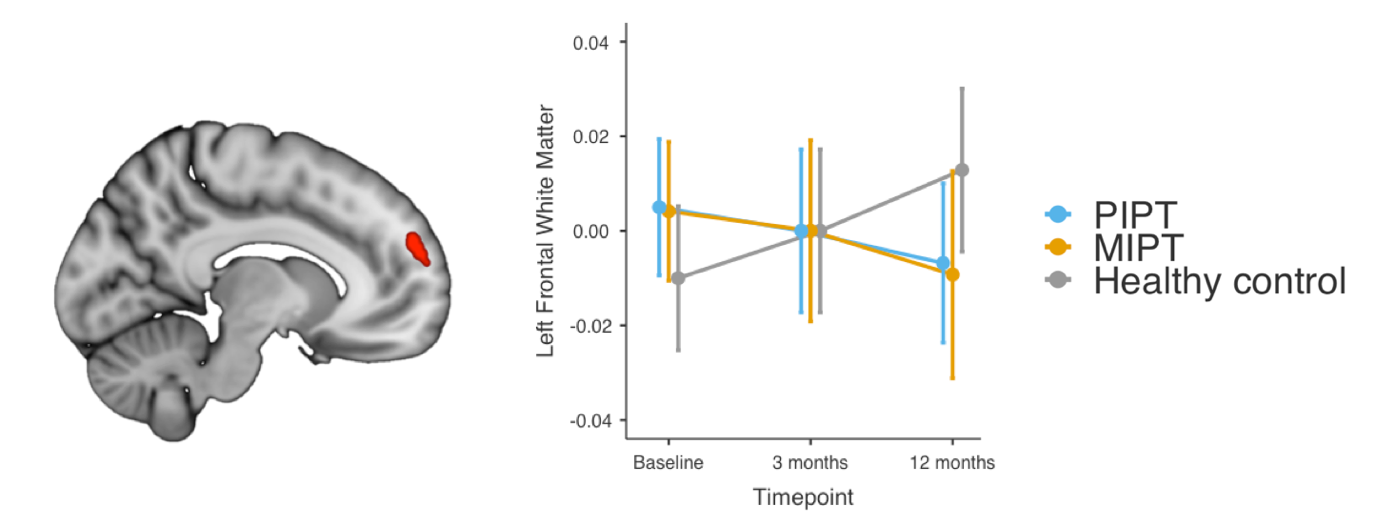


Supplementary Figure 6 – Red clusters indicates frontal white matter where differences in linear trend (p < 0.001, uncorrected) were detected between baseline and 12 months.
